# The impact of the Systematic Assessment for Resilience (SAR) framework on students’ resilience, anxiety, depression, burnout, and academic-related stress: a quasi-experimental study

**DOI:** 10.1186/s12909-024-05444-9

**Published:** 2024-05-07

**Authors:** Majed Wadi, Ali Shorbagi, Sarra Shorbagi, Mohamed Hassan Taha, Muhamad Saiful Bahri Yusoff

**Affiliations:** 1 Medical Education Department, College of Medicine, Qassim University, Buraydah, Saudi Arabia; 2 Department of Clinical Sciences, College of Medicine, University of Sharjah, Sharjah, United Arab Emirates; 3 Department of Family and Community Medicine and Behavioral Science, College of Medicine, University of Sharjah, Sharjah, United Arab Emirates; 4 College of Medicine and Medical Education Center, University of Sharjah, Sharjah, United Arab Emirates; 5 Medical Education Department, School of Medical Sciences, Universiti Sains Malaysia, Kubang Kerian, Malaysia

**Keywords:** Resilience, Assessment, Medical students, Medical Education, Stress, Anxiety, Burnout, Depression

## Abstract

**Background:**

Medical students face significant psychological stress, impacting their academic performance and well-being. The Systematic Assessment for Resilience (SAR) framework is designed to enhance resilience and mitigate stress among medical students, addressing the need for interventions within the assessment system in medical education. The aim of this study was to evaluate the implementation of SAR framework on medical students’ resilience, anxiety, depression, burnout, and academic stress.

**Methods:**

This study employed a quasi-experimental design with pre- and post-testing. It involved the training of course coordinators in implementing the SAR framework and its integration into the daily learning activities. Fourth-year medical students were assessed before and after the intervention using standardized measures of resilience, anxiety, depression, burnout, and academic stress. Data were analyzed using quantitative methods and thematic analysis for qualitative feedback.

**Results:**

Post-intervention, students demonstrated a significant increase in resilience scores (*p* < 0.001) and a notable decrease in measures of anxiety, depression, and academic stress (*p* < 0.001). The burnout types were also statistically different (*p* < 0.001) except client-related burnout (*p* > 0.05). Qualitative feedback of the course coordinators highlighted an improved learning environment, increased coping strategies, and a more supportive academic culture.

**Conclusion:**

The SAR framework significantly contributes to enhancing medical students’ resilience and reducing psychological distress. Its implementation suggests a promising approach to fostering a supportive educational environment that not only addresses the psychological challenges faced by medical students but also enhances their academic performance and overall well-being. Further research is warranted to explore the long-term impacts of SAR across different medical education contexts.

**Supplementary Information:**

The online version contains supplementary material available at 10.1186/s12909-024-05444-9.

## Introduction

Medical students perceive medical school as a demanding environment due to the nature and magnitude of the curriculum’s contents [1] and the pressure of numerous assessments that influence important decisions in their lives [2]. Systematic reviews and meta-analyses reveal alarming figures regarding the extent to which medical students suffer from stress and its consequences, such as anxiety and depression [3–5]. It has been found that 30% of medical students suffer from stress, anxiety, or depression [5–7]. Other studies have reported even higher rates of these problems (70%) [8, 9]. Chronic stress aggravates other undesirable problems, such as poor academic performance, poor peer relationships, academic dishonesty, depression, and even sleep and eating disorders [10, 11]. Additionally, chronic stress is connected to drug addiction, alcoholism, and suicide [3–5]. It has been found that doctors who suffer stress throughout their undergraduate education may develop a negative attitude toward their profession that could threaten patient care [5–7].

Based on these facts, it is sensible to identify the causes of stress and to implement interventions to alleviate this burden. Multiple studies have found that tests and exams are among the most identified causes [4, 5, 12, 13]. Consequently, several interventions have been implemented to combat what is known in the literature as test anxiety [7, 14]. In their systematic review, Soares and Woods [15] summarized several studies on test anxiety reduction and found a lack of collaboration between the identified interventions and the school site, which impeded the maximum benefit of intervention and the desired outcomes. Furthermore, the identified studies focus on a single aspect of the problem without addressing a solid theoretical framework or a holistic perspective on other factors that influence the situation [15]. Consequently, it has become vital to seek comprehensive approaches that incorporate both individual and systemic (organizational) factors [4, 5]. With the expansion of resilience research, medical educators are increasingly interested in identifying interventions that foster resilience among medical students [3, 16, 17].

Resilience is a construct that cultivates a variety of characteristics necessary for adapting to the dynamic changes of life and maintaining well-being [18]. Resilience is no longer viewed as a set of inherited traits that enable an individual to overcome life’s challenges and adversities; rather, it is viewed as a dynamic process and an acquired trait, meaning that it can be taught and fostered [19, 20]. Despite varying definitions of resilience, the general and agreed definition is the individual’s capacity to deal with and overcome adversity appropriately and effectively that boosts well-being [4, 18, 21–24]. Accordingly, resilient students are those who can maintain their abilities and continue to grow despite academic and life obstacles [3, 25–27]. According to related literature, resilience can be fostered by working on multiple levels. The first level begins with internal factors, which means advancing the psychological process in the face of adversity and enhancing what are called “internal traits” [28]. The second level involves exposing individuals to simulated adversities and providing them with opportunities for reflection [21, 29]. The third level derives from the extent of institutional or organizational efforts to provide protective factors and fertile resources (external protective factors) to equip individuals with the necessary protective factors to overcome life’s adversities [28, 30].

In their recent research, Wadi et al. developed a framework for fostering resilience through the process of student assessment [31]. This framework is called the Systematic Assessment for Resilience (SAR) framework and encompasses numerous guidelines for fostering resilience and enhancing its four constructs: (1) self-control, in which students should be able to govern themselves and face adversity; (2) management, which describes the students’ ability to use available resources effectively to overcome obstacles; (3) engagement, which highlights the students’ ability to be involved and committed to pursuing challenges with perseverance; and (4) growth, which reflects the students’ ongoing development to face future challenges. The four constructs work together in a cycle to promote resilience through five phases of assessment: (i) direction, which focuses on improving the candidate’s understanding of the assessment’s scope and procedure; (ii) preparation, which emphases enhancing candidates’ cognitive, mental, and psychomotor readiness to optimize assessment performance; (iii) experience, which helps enhance the formative assessment component; (iv) examiner focus, which deals with improving examiner behavior to increase candidate performance and decrease candidate anxiety; and (v) student reflection, which encourages self-review [31]. The SAR framework presents a comprehensive approach that promises to foster a learning environment in medical schools that supports mental well-being and cultivates resilience in medical students. Therefore, this study aims to evaluate the impact of the SAR framework implementation on enhancing resilience and reducing anxiety, depression, and academic-related stress among medical students.

## Methods

### Study setting

The study was conducted at the University of Sharjah, College of Medicine (UoS-CoM), which offers a six-year MBBS program delivered in three phases: Phase I is the foundation year and Phase II is the pre-clerkship phase, which includes years 1, 2, and 3. This is followed by Phase III, a clerkship phase, that comprises years 4 and 5. In the clerkship phase, students receive clinical training in various departments in public and private hospitals in Sharjah. Year 4 consists of four clinical rotations (10 weeks each) in the four major divisions of Medicine, Surgery, Pediatrics, and Obstetrics & Gynecology, while in Year 5, the rotations include medicine and surgery sub-specialties such as Neurology, Dermatology, Cardiology, Nephrology, ENT, and Ophthalmology, as well as Family Medicine and Psychiatry [32].

### Study population

1. Students.

For logistical considerations, Year 4 students were selected as study participants. All 149 fourth-year medical students registered for the academic year 2021–2022 at UoS-CoM were invited to participate in this study.

2) Course coordinators.

Eight Year 4 course coordinators, responsible for planning courses, organizing educational events, facilitating bedside training in teaching hospitals, and managing learning materials in the learning management system, were invited to participate in the study.

### Eligibility criteria of participants

For students, only those who participated in both the pre- and post-stages of the study will be included in the analysis of the results. As for course coordinators, only those who agree to participate in the in-depth interview will be included in the analysis of results.

### Study design

The study used a quasi-experimental design with one group pre- and post-test [33]. The decision to opt for a single-group design was driven by ethical considerations, as randomizing student participants into intervention and control groups posed ethical concerns related to potential unequal benefits [33]. Additionally, the feasibility of maintaining the integrity of training materials among course coordinators in the target group further supported the choice of a single-group pre-test and post-test design [34].

**Fig. 1:**
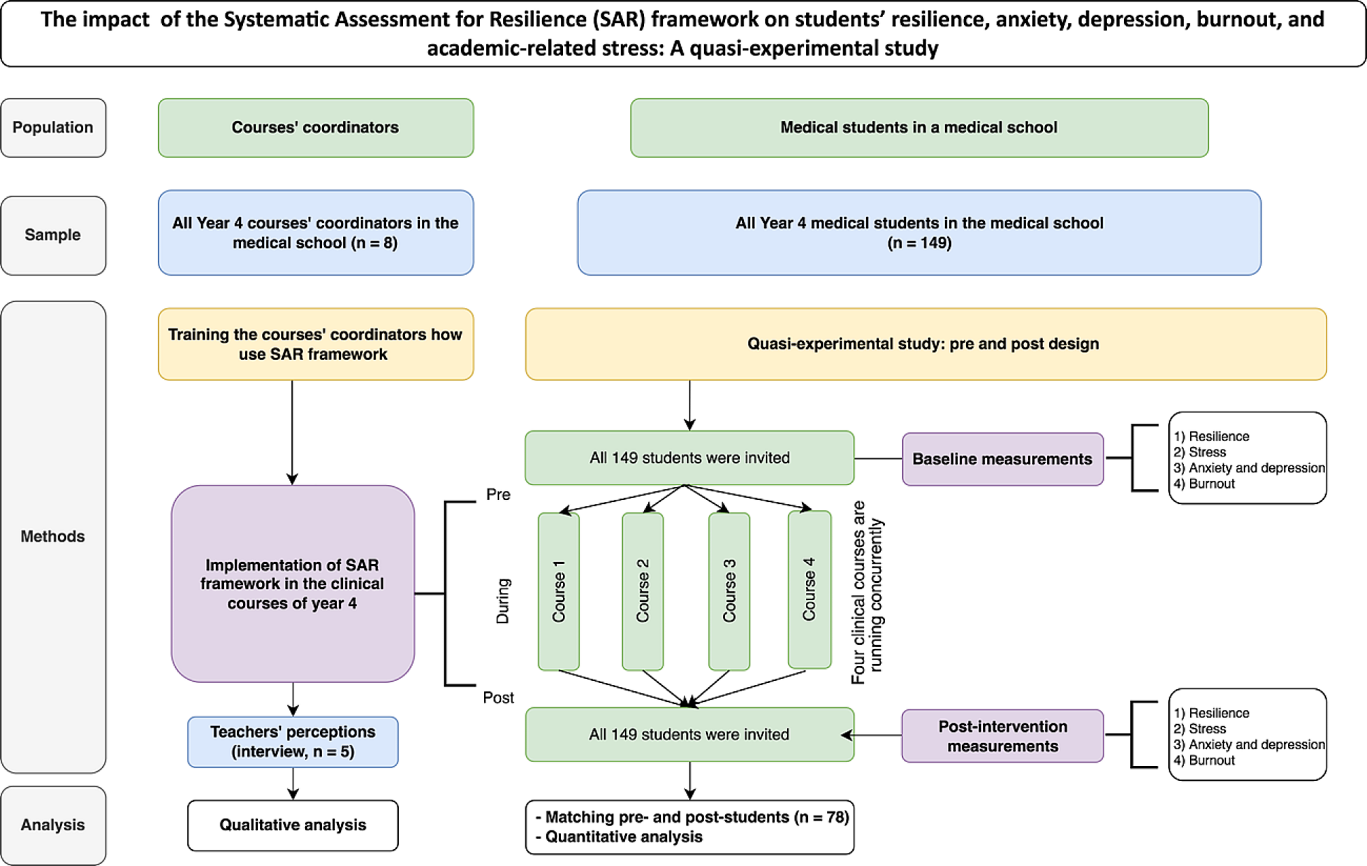
Study flowchart

The design begins with training all course coordinators on how to use the SAR framework as a daily practice in their respective clerkships and then measuring its effect on students before and after its implementation. Furthermore, in-depth interviews were conducted with the course coordinators (Fig. 1). The purpose of this qualitative phase was to gain insights into the perceptions of the SAR guidelines among medical teachers, as well as to assess the feasibility and applicability of these guidelines in the clinical setting.

### Study intervention

The intervention began with a training session on how to utilize and implement the SAR framework, which was completed before the clerkship rotation began. It was a five-hour online training workshop provided by Microsoft Teams for fourth-year medical educators (Appendix I). The course coordinators were informed that SAR consists of guidelines that promote resilience throughout the various phases of assessment, from planning to implementation to evaluation. Participants were given a group activity consisting of a list of SAR guidelines presented in the form of a series of yes-or-no questions regarding how to apply the SAR guidelines (Appendix II). To facilitate the effective implementation of the guidelines, they were tabulated, and each was accompanied by an example of how and when to use it (Appendix III). In addition, the participants were supported/monitored through ongoing online communication (WhatsApp group, online meetings, email reminders) to discuss and clarify any concerns regarding SAR guidelines. They were not required to implement all of the framework’s guidelines; rather, they were free to decide which guidelines were feasible and applicable to their clerkships. As a result, all students received the intervention, which was tailored based on the guidelines implemented by their course coordinators.

### Data collection

#### Quantitative data

Quantitative data were gathered from Year 4 medical students whose course coordinators implemented the SAR guidelines in their respective courses. Before starting their respective clerkship rotations, all students were invited to complete the study’s measurement instruments through a Google form, distributed to them via their official email addresses. Four tools were used: (1) the Medical Professionals Resilience Scale (MeRS) to measure resilience, (2) the academic-related stressors (ARS) part of the Medical Students Stressor Questionnaire (MSSQ), (3) the depression and anxiety parts of the Depression and Anxiety Stress Scale (DASS), and (4) the Copenhagen Burnout Inventory (CBI). Appendix IV contains full details of study tools and how they were scored, however, a general overview of these tools is described below.

The Medical Professionals Resilience Scale (MeRS) [35] measures four domains of resilience based on the integrated resilience model [36]: control, resourcefulness, involvement, and growth dimensions. The MeRS is a valid and reliable scale with a Cronbach’s alpha of 0.914 [35]. It presents 37 items to rank according to a four-point Likert scale (1 *Strongly disagree*, 2 *Disagree*, 3 *Agree*, and 4 *Strongly agree*). Six items assess the domain of control, which refers to medical professionals’ ability to remain calm and composed in the face of adversity, four assess resourcefulness—the ability to use available resources to overcome adversity—twelve assess commitment, and fifteen assess growth capability and resilience following adversity.

The Medical Student Stress Questionnaire (MSSQ) [37] contains 40 items covering six dimensions of stress unique to medical students—academic stressors, interpersonal stressors, teaching and learning stressors, social stressors, drive/desire stressors, and group activity stressors. The questionnaire is based on a four-point Likert scale and has a good level of validity and reliability, with a Cronbach’s alpha of 0.95 in the initial study [37], and its reliability has been consistently upheld across various contexts and cultures [38, 39]. For the purpose of this study, only the items measuring academic-related stressors (ARS) were used.

The Depression, Anxiety, and Stress Scale (DASS-21) [40] is a widely used screening tool that utilizes a tripartite approach to assess three dimensions of emotional states: depression (low positive affect), anxiety (physiological hyperarousal), and stress (negative affect) [41]. Each dimension of the DASS-21 contains seven items. The respondents were asked to consider how much each statement applied to them in the previous week and rank each statement accordingly. A score of 0 means *Did not apply to me at all*, 1 = *Applied to some degree or some of the time*, 2 = *Applied to me to a considerable degree or a good part of time*, 3 = *Applied to me very much or most of the time*). DASS-21 has an overall Cronbach’s alpha of 0.93 [40] in the original study. This reliability has been mirrored in its global application [42].

The Copenhagen Burnout Inventory (CBI) [43] is a self-reporting inventory comprised of 19 items that assess personal-, work-, and client-related burnout. The personal-related burnout domain has six items and assesses respondents’ levels of physical and psychological fatigue or exhaustion independent of their work. The work-related burnout domain contains seven items and assesses respondents’ levels of physical and psychological fatigue or exhaustion as a result of their employment. The client-related burnout domain has six items and assesses the respondent’s bodily and psychological weariness or fatigue as a result of their clients. Client is a broad term that encompasses individuals, such as students, customers, and patients. In accordance with the CBI guide, the phrase “client-related burnout” should be used when referring to the sample’s participants, and this domain will henceforth be referred to as shown. It has been confirmed using Cronbach’s alpha values of 0.85 to 0.87 for each domain [43], the reliability of the instrument has been consistently evident in various studies utilizing the CBI [44].

For the purpose of analyzing and matching students’ responses in pre- and post-intervention, students were asked to include a distinct number in both surveys to facilitate the matching of pre- and post-intervention responses without compromising confidentiality.

### Qualitative data

Qualitative data were collected at the end of the clinical courses via in-depth interviews with the course coordinators. The interview was conducted in person and recorded for verbatim transcription and analysis. Figure 1 is a flow chart illustrating the steps of the study.

### Data analysis

#### Quantitative data

Initially, each participant’s pre- and post-intervention data were matched, and descriptive and inferential analyses were conducted using SPSS 23 with a 95% confidence interval and a significance level of 0.05. Based on the majority of measurement tools, the responses were calculated as means and standard deviations (SD). The paired t-test was used to compare the means of pre- and post-intervention results among students. McNemar test was used to analyze the difference of burnout cases pre- and post-intervention. Additionally, the Pearson correlation coefficient was used for pre- and post-intervention measurements to evaluate study parameters.

### Qualitative data

Braun and Clark’s six-phase thematic analysis method was used for the analysis of the transcripts of the interviews [45]. In the first step (data familiarization phase), the authors familiarized themselves with the data transcribed verbatim from the audio recordings, assigned pseudonyms to all identifiable individuals, and cross-checked the transcript against the audio recordings. In the second step (generating initial codes), the authors imported the transcript into Atlas.Ti (version 7.9) and began identifying open codes across the dataset, including the participants’ own words (in vivo) or a descriptor for their experience. Next, in the third step (searching for themes), the authors sought themes by combining several related codes to generate overarching themes. Then in the fourth step, reviewing the themes, the authors reviewed the themes and their coherence with the related quotes and defined and named the themes (fifth step) to write the report. Finally, as the sixth step, the authors wrote the report.

To ensure the trustworthiness of qualitative data, the researchers employed Guba’s four criteria: credibility, transferability, dependability, and confirmability [46]. Credibility (akin to internal validity) was enhanced through prolonged engagement during interview, ensuring participants fully expressed their experiences, and member checking, which involved validating summaries of discussion with participants “on the spot”. Transferability (comparable to external validity) was facilitated by providing detailed descriptions in the methodology section of the study’s context and participants, enhancing the applicability of findings across different settings. Dependability was secured through a code-recode strategy, involving periodic reevaluation of data coding to verify consistency. Lastly, confirmability was established by creating an audit trail, documenting the research process and analysis, thus offering a transparent record for external examination.

## Results

After matching students’ pre-and post-intervention responses, 78 students of 149 had participated in both phases. The characteristics of the students are shown in Table 1.

**Table 1:** Student demographics (*n* = 78)

Variables	
**Gender**, ***n*** (%)	
Male	22 (28.2%)
Female	56 (71.8%)
**Age, mean (SD)**	
Male	21.90 (0.92)
Female	21.96 (0.57)

Pre- and post-intervention scores of the measured parameters were compared for all students. Collectively, the mean differences in resilience (as a global score), as well as its underlying four constructs, showed negative values, indicating a significant increase in these parameters after the intervention, whereas the difference in the mean scores of depression, anxiety, and academic-related stressors showed positive values, indicating a significant reduction of these parameters after the intervention. There were statistically significant differences between the mean scores for all parameters (Table 2).

**Table 2:** Comparison of means of study variables pre- and post-intervention for all students

Measurements	Study stage	Mean (SD)	Mean difference (95% CI)	t-statistics (df)	*p*-value*
**Global score of resilience**	Pre	108.97 (19.47)	-9.62 (-12.82, -6.41)	-5.97 (77)	< 0.001
	Post	118.59 (23.30)			
**Growth domain of resilience**	Pre	47.78 (8.46)	-3.26 (-4.72, -1.79)	-4.43 (77)	< 0.001
	Post	51.04 (9.59)			
**Control domain of resilience**	Pre	34.14 (8.02)	-3.60 (-4.79, -2.42)	-6.05 (77)	< 0.001
	Post	37.74 (9.51)			
**Involvement domain of resilience**	Pre	17.63 (4.35)	-1.74 (-2.41, -1.07)	-5.19 (77)	< 0.001
	Post	19.37 (5.00)			
**Resourceful domain of resilience**	Pre	9.42 (3.17)	-1.01 (-1.36, -0.66)	-5.77 (77)	< 0.001
	Post	10.44 (3.49)			
**Depression**	Pre	23.36 (13.03)	4.92 (3.13, 6.71)	5.48 (77)	< 0.001
	Post	18.44 (10.05)			
**Anxiety**	Pre	22.64 (13.47)	5.51 (3.58, 7.45)	5.68 (77)	< 0.001
	Post	17.13 (9.68)			
**Academic-related Stressors (ARS)**	Pre	2.19 (0.81)	0.25 (0.15, 0.35)	5.16 (77)	< 0.001
	Post	1.94 (0.64)			

*Paired t-test was applied

The analysis of student burnout pre- and post-intervention revealed significant shifts in two categories of burnout (Table 3). For personal-related burnout, all 21 participants initially classified as non-cases remained unchanged, highlighting the intervention’s stability in non-affected individuals. Conversely, 12 participants previously identified as cases transitioned to non-cases, showcasing a notable positive change (*p*-value < 0.001). In the realm of work-related burnout, while 25 non-cases maintained their status, 11 cases shifted to non-cases, underscoring the intervention’s effectiveness (*p*-value = 0.001). However, in client-related burnout, although a shift was observed with 6 moving from case to non-case, this change was not statistically significant (*p*-value = 0.289) (Table 3).

**Table 3:** Comparison of burnout pre- and post-intervention for all students

**Type of burnout**	**Pre-intervention**		**Post-intervention**		***p***-**value**
			Non-case	Case	
**Personal-related burnout**		Non-case	21 (26.9%)	0	< 0.001*
		Case	12 (15.4%)	45 (57.7%)	
**Work-related burnout**		Non-case	25 (32.1%)	0	0.001*
		Case	11 (14.1%)	42 (53.8%)	
**Client-related burnout**		Non-case	52 (66.7%)	2 (2.6%)	0.289*
		Case	6 (7.7%)	18 (23.1%)	

*McNemar test was used

The correlation matrix presented in Table 4 reveals substantial associations among the study variables pre- and post-intervention. Global resilience and its four domains consistently showed strong, positive correlations in both assessments. Depression, anxiety, and academic-related stress (ARS) were positively correlated with various burnout types. Inversely, global resilience and its subcomponents negatively correlated with these psychological stress indicators, suggesting that higher resilience is linked to lower levels of depression, anxiety, and stress.

**Table 4:** Correlation of study measurements in the pre and post-intervention stages

Stage of study	Measurements	Score of Global Resilience	Score of Growth construct	Score of Control construct	Score of Involvement construct	Score of Resourceful Construct	Score of depression	Score of anxiety	Score of academic-related stressors	Personal-related burnout	Work-related burnout	Client-related burnout
Pre-intervention	Score of Global Resilience	1										
	Score of Growth construct	0.887*	1									
	Score of Control construct	0. 947*	0. 793*	1								
	Score of Involvement construct	0. 667*	0. 355*	0. 618*	1							
	Score of Resourceful Construct	0. 465*	0. 282*	0. 320*	0.215*	1						
	Score of depression	-0. 313*	-0.81	-0.248*	-0.558*	-0.313*	1					
	Score of anxiety	-0. 208	-0.109	-0.158	-0.396*	-0.042	0.844*	1				
	Score of academic-related stressors	-0. 215	-0.098	-0.190	-0.284*	-0.191	0.531*	0.528*	1			
	Personal-related burnout	-0.195	-0.616	-0.058	-0.305*	-0.129	0.672*	0.672*	0.496*	1		
	Work-related burnout	-0. 431*	-0.279*	-0.438*	-0.447*	-0.178	0.623*	0.579*	0.595*	0.800*	1	
	Client-related burnout	-0.163	-0.110	-0.101	-0.156	-0.235*	0.356*	0.375*	0.731*	0.552*	0.615*	1
Post-intervention	Score of Global Resilience	1										
	Score of Growth construct	0.918^*^	1									
	Score of Control construct	0.953^*^	0.836^*^	1								
	Score of Involvement construct	0.780^*^	0.577^*^	0.721^*^	1							
	Score of Resourceful Construct	0.441^*^	0.279^*^	0.312^*^	0.228^*^	1						
	Score of depression	-0.215	-0.073	-0.205	-0.346^*^	-0.183	1					
	Score of anxiety	-0.123	-0.078	-0.128	-0.167	-0.018	0.862^*^	1				
	Score of academic-related stressors	-0.005	0.108	-0.094	-0.068	0.024	0.558^*^	0.594^*^	1			
	Personal-related burnout	-0.222	-0.165	-0.235^*^	-0.181	-0.125	0.679^*^	0.649^*^	0.508^*^	1		
	Work-related burnout	-0.388^*^	-0.312^*^	-0.433^*^	-0.331^*^	-0.084	0.656^*^	0.640^*^	0.567^*^	0.880^*^	1	
	Client-related burnout	-0.240^*^	-0.206	-0.206	-0.174	-0.227^*^	0.407^*^	0.422^*^	0.549^*^	0.686^*^	0.685^*^	1

*Correlation is significant at the 0.05 level

### Qualitative analysis of in-depth interviews

In-depth interviews were conducted with five course coordinators who agreed to participate to offer their perceptions of the SAR framework and its guidelines. Interviews were conducted through an online platform, each lasting around 35 min. The qualitative analysis of the interviews revealed the themes shown in Table 5. Generally, all coordinators acknowledged the feasibility of implementing the SAR framework with their educational practices; for example, briefing the students about assessments, sharing the assessment rubrics with the students, implementing formative assessment and mock exams, providing supporting material about coping and study skills, encouraging peer and self-assessment, and orienting the examiners to create a less stressful environment during the exams. Concerning observed effectiveness, the coordinators stated that they noticed that students in the intervention were generally self-assured and were enjoying the rotation compared to other batches. Regarding the challenges faced with the SAR after its implementation, one coordinator expressed that some strategies could not be done without official approval from the curriculum committee; for instance, introducing collaborative assessment and open book exams. Almost all the coordinators emphasized that components included in the SAR framework should be obligatory as part of educational practices.

**Table 5:** Themes and quotes of course coordinators

Theme	Sub-theme (if any)	Examples of Quotes
**Feasibility of SAR**		*Initially, I did not think that it would be feasible, but now I believe that it is.* (Course coordinator 1)
**Implemented strategies**	Assessment briefing	*What I tried to do actually to follow the model is just to make it more organized and structured. So, for example, briefing and familiarizing students with different modalities of the assessment.* (Course coordinator 2)
	Assessment rubric	… *We also shared some rubrics.* (Course coordinator 2)
	Formative assessment	*We give them a lot of MCQ before our case-based discussion, and we go through the discussion on the learning point.* (Course coordinator 3)
	Mock exam	*We added a mock DOCEE (direct observation of the clinical encounter examination) … so they learned and will have less stress when they go through the real DOCEE.* (Course coordinator 4)
	Case write up	… *I did a session with all of them. It’s like a simulation session where we start with a case and simulate how it should be written, and the students were coming up with how they would try it… There was a marked difference in the performance of the whole team, and I believe this is because they are more aware of these tips.* (Course coordinator 5)
	Simulation	…*We did a simulated case where I explained everything to them. I explained how they should proceed, the systematic approach to it.* (Medical teacher 4)
	Study skills for exam	*After we saw these videos, we tried to implement them. We talk to students about exam-taking strategies, how to answer questions, and how to use their time.* (Course coordinator 5)
	Coping skills	*I actually shared some links with the students which will be helpful to them. I sent them these links through Blackboard and explained that these links are important for you. You need to go through them, and they may help you reduce the stress and manage your time, and we will help you with your preparation for the exam.* (Course coordinator 2)
	Training of clinical examiners	*We have conducted training sessions through a faculty development program where we meet with all the faculty (from hospitals) who are involved with our students, I mean teaching and assessment. So, part of that program is talking about the examination and its environment, and how we could help the students reduce stress.* (Course coordinator 2)
	Introducing humor before exam	*We were making jokes and telling the students funny stories. We also said “congratulation” beforehand to encourage them…* (Course coordinator 4)
	Peer assessment	*The students reflect on their own learning. For example, one student goes and does the emergency scenario and the other students comment and give him feedback, and he (himself) gives feedback about himself. This goes around for all the students.* (Course coordinator 3)
	Students’ feedback	*In the DOCEE, we sit with the students and give them feedback. We say, ‘this is your area of strength. This is the area you probably need to focus on for the future.’* (Course coordinator 4)
**Observed effectiveness**	Reduced anxiety	*I personally find this (sharing rubric) is really very helpful. When we were very transparent with the students about the rubric, they were really very reassured, and they kind of felt that they don’t have to worry about who their examiner is. They were very reassured by this, and we are definitely going to make this a practice.* (Course coordinator 2)
	Enjoying the learning	*I noticed that the students liked the rotation better than the previous batch had. They told us that they benefited from these strategies that we gave them via Blackboard. They commented on how organized the rotation was.* (Course coordinator 4)
	Improved performance	*Application of SAR has a very positive impact on the students; we noticed that after the rotation in which we applied it, the scores of the students improved. They scored much higher than the 1st and 2nd rotations.* (Course coordinator 4)
	Self-regulated learning	*Application of SAR strategies made the students realize where their knowledge has a gap, where they need to fill it, and how to fill it. Yeah, it just gave them a road map to know how to proceed with their self-education.* (Course coordinator 5)
	Improved well-being	*SAR definitely improves the psychological well-being of the students and accordingly of the faculty themselves.* (Course coordinator 5)
**SAR prospective**	Part of the curriculum	*Incorporating SAR strategies within the curriculum is important.* (Course coordinator 1)
	Top managerial support	*Implementation of all these strategies should be done, and the official process should start from the curriculum committee and the assessment committee.* (Course coordinator 2)
	Obligatory training	*I think the model is really good and effective. We need an obligatory course to make every one of us involved in the teaching or the assessment aware of these strategies.* (Course coordinator 2)

## Discussion

In contrast to other resilience interventions aimed at enhancing resilience at the individual level that use interventions with a limited perspective, the current study utilized a holistic approach to foster resilience as an integral part of daily assessment practices among medical students in their clinical years. Consequently, the purpose of applying the SAR framework is to sustain and foster resilience as part of the organization’s educational process [17, 47]. The efficacy of the intervention was verified by measuring its impact on students’ psychological parameters (resilience, academic stress, anxiety, depression, and burnout), as well as by ascertaining the perspective of medical educators who used the framework. The analysis of psychological measurements yielded statistically significant results, and the qualitative analysis of the educators’ perspectives reported positive feedback. Below, we discuss the results based on study measurement tools and support the quantitative results with qualitative findings obtained from the participating educators.

### Resilience

The current study revealed that applying the SAR framework improved resilience significantly, reflecting the dramatic effect of SAR as an intervention. These findings can be explained from three aspects: (i) the approach of the SAR intervention, (ii) the nature of the guidelines, and (iii) the specific resilience measurement tool.

The SAR intervention approach is holistic and integrative. It incorporates resilience principles at multilevel steps across different stages of assessment, while simultaneously employing various preventive measures to counteract the opposite effect of anti-resilience constructs, particularly anxiety and stress. Indeed, the SAR framework utilized Huey and Palaganas [3] recommendation to combine organizational and individual factors to promote resilience in an HPE context. Furthermore, SAR promotes resilience at all stages of assessment: before, during, and after. The SAR guidelines aim to improve students’ protective factors during pre-assessment so that they can pursue the assessment with adequate resources and preparation. It has been demonstrated that providing students with adequate protective factors improves resilience [48]. During the assessment, the SAR guidelines aim to gradually expose students to uncertainty and difficulty through formative assessment or mock exams, so that they will engage in adversity but with support (feedback) to help them overcome difficulties and achieve their goals [49, 50]. They will eventually develop confidence and belief in their own abilities (self-efficacy) [51, 52]. As a result, they will be able to face similar struggles in the future [36]. The SAR framework provides opportunities for self-reflection following assessment. Students evaluate their performance based on constructive feedback, reformulate their goals [52, 53], and benefit from the experience (growth and transformation) to face future challenges [53].

The nature of SAR guidelines is to make them part of daily practice as checklists facilitating their use. Checklists act as reminders of the most crucial steps that even highly qualified professionals might forget [54]. It has been demonstrated that using checklists is an easy and effective way to boost both individual and group performance [55]. Besides using checklists, SAR provides fertile resources explaining how every guideline can be applied. The courses’ coordinators acknowledged that they were pleasantly surprised by the way SAR could be integrated into assessment practice. They also commented on the richness of the provided resources, which facilitated the use of SAR in their practice, and the positive feedback they received from students (Table 5). Therefore, SAR can be described as a proactive and supportive framework for promoting resilience [56].

The last point supporting these findings was the result of a specific resilience measurement tool, the Medical Professionals Resilience Scale (MeRS) [35]. This tool was created based on the integrated resilience model [57]. The MeRS has specific items measuring a particular resilience construct. Even though this tool was validated for practicing doctors [35], the uniqueness of the items in measuring four resilience constructs—control, resourcefulness, involvement, and growth—supported its use in the current study. Several systematic reviews have recommended that using a specific measurement tool in resilience intervention is crucial for validating the intervention’s effectiveness [19, 58, 59].

Contrasting the current findings with previous research in terms of study design and the relationship between assessment and resilience is worthwhile. In terms of using an experimental design, the current study is comparable with certain studies identified in the systematic review that was done by Cleary, Kornhaber [58] in which numerous interventional studies using single-arm pre- and post-intervention measurement were very effective in promoting resilience among health professionals, and comparable with the findings of Kunzler, Helmreich [59] meta-analysis which found that several randomized controlled trials were very effective in promoting resilience among health professionals.

Regarding those studies’ attempts to explain the relationship between assessment and resilience, Berg and Pietrasz [60] used experiential classroom exercises (competitive activities) to help students develop resilience. Similarly, Clipa et al. [16] found that students’ resilience increases when they practice formative assessment, which reduces test anxiety. Although both studies have the same findings, the intervention proposed in the current study was developed based on a solid evidence-based framework [31].

The current findings are consistent with those of Liu et al. [61], who found that resilience mediated the relationship between emotion regulation and test anxiety and discovered that developing resilience improves emotional regulation, which automatically lowers test anxiety. The current study used SAR as an intervention not only to promote resilience but also to enhance emotional regulation. The employed approach targeted individual and environmental levels at various stages of assessment: pre-, during, and post-assessment to improve emotion regulation. The SAR contains guidelines focusing on emotion regulation, such as “advising students on exam skills” and “providing strategies for students to reduce test anxiety.” These guidelines principally aim to mitigate anxiety, thus fostering resilience. Cobbinah and Amoako [62] used a non-experimental study design to show that the assessment promotes resilience by causing students to manage and cope well with academic stress caused by “the assessment” without support and guidance, whereas the current study used an empirical study design (quasi-experimental) and designed a comprehensive assessment framework to promote resilience proactively. The current study provided protective factors (resources for assessment direction and preparation). These resources serve as enablers for achieving resilience and equip students with the resources they need to face adversity. In the absence of these resources, resilience will not grow. This works in the same way that adequate nutrition does after vaccination. Nutrition strengthens the immune system in the face of the vaccine [50, 63].

### Academic-related stress

The implementation of SAR significantly reduced academic stress, reflecting the effectiveness of the framework in improving psychological health. Obtaining this result may be due to the approach of the SAR framework. It adopts the implications of the transactional stress model of Lazarus and Folkman [64] and incorporates sets of guidelines at various levels to counteract stress and nurture the coping mechanisms of medical students. According to this model [64], there are three stages of stress development: (1) primary appraisal, influenced by stress antecedents; (2) secondary appraisal, influenced by stress mediators; and (3) reappraisal, influenced by stress consequences. Primary appraisal is the process by which an individual evaluates an event and decides whether it is positive, threatening (negative), or irrelevant [64]. This is influenced by personality traits (such as social evaluative trait anxiety, self-efficacy, and optimism) and environmental factors (such as the test atmosphere, the amount of social support available, and the nature of the cognitive task) [65]. The SAR framework encourages the personal traits and environment toward the positive side. It enhances self-efficacy and optimism through the frequency of formative assessment and feedback so that the students will acquire self-regulatory learning, which in turn enhances self-efficacy and optimism. Regarding the environment, it guides course coordinators to enrich the test environment with multiple factors to reduce the sources of test anxiety. The secondary appraisal appears if a negative event occurs. In this phase, the process starts when a person evaluates the resources available to deal with a situation. When a loss occurs, harm appraisal occurs. When a person anticipates harm, threat appraisal occurs; when they are confident in their ability to meet the demand of an event, challenge appraisal occurs [64]. Zeidner [65] described this psychological process as a stress mediator. The SAR framework was designed to empower the psychological process (stress mediators) so that the individual will have enough ability to appraise the harm event and overcome the loss if it occurs. This characteristic is the core of resilience [19, 30]. Reappraisal occurs when new information or resources for dealing with an event become available. It is a continuous reevaluation of the event [64, 66] influenced by individual characteristics and coping strategies [65]. The SAR is also designed to provide avenues for reappraisal and to strengthen personal characteristics and coping strategies in advance.

Another point to elucidate the findings is that the stress measurement instrument is primarily focused on academic-related stressors (ARS). ARS items are very specific to stressors arising from the academic context [37]. Because the SAR framework aims to promote resilience and simultaneously counteract other anti-resilience factors such as academic stress, the results are expected. The result is comparable with the randomized controlled trial (RCT) conducted by Yusoff and Esa [67], in which the same tool was used but the intervention was different. It was a workshop enhancing coping skills in the face of adversities. The current study utilized a holistic approach through daily practice of assessment and curriculum delivery. The SAR framework provides a range of advice on how to overcome test anxiety, how to manage time, and how to study properly (Appendix III). All these skills were utilized as system-built approaches not only to foster resilience but also to enhance the protective factors of students and enhance medical teachers’ ability to provide a safe and healthy academic environment [48]. Notably, the medical educators reported a remarkable change in the students’ behavior exemplified by their favorable attitude toward the academic environment (Table 5).

Comparing the current findings with those of other studies that used resilience-focused intervention demonstrates that resilience intervention has an enduring effect on stress that can persist for six months after intervention [19, 58, 59, 68]. The current study adds to this body of evidence by providing another empirical finding to support the nexus between reducing academic stress and improving resilience through assessment practice.

Nevertheless, the current study contradicts the findings of Lo et al. [69], who found that psychological interventions did not affect stress but that cognitive-behavioral, relaxation, and mindfulness interventions may improve the mental health of health profession students. The current study combines psychoeducation and cognitive interventions by providing students with useful links to a variety of resources based on these approaches and advising them to use them.

### Anxiety

A significant reduction in anxiety symptoms was observed in the current study. This was not unexpected, as the intervention incorporates multiple components that not only reduce test anxiety and the negative impact of assessment but also reduce other anxieties arising from the burden of studying medicine, while simultaneously fostering self-efficacy, a strong predictor of resilience [51, 70].

The guidelines provide medical educators with several practical tips for students to overcome academic challenges, including exam-taking strategies and self-care techniques, as well as advice on reducing test anxiety [71].

The current findings provide additional evidence for earlier systematic reviews [3, 58, 59] that discovered a significant effect of resilience interventions in lowering anxiety among HPE students and practitioners. The current study, however, diverges from the findings of Lo et al. [69], who found that while cognitive-behavior, relaxation, and mindfulness interventions may improve the mental health of health professional students, psychoeducational interventions did not affect stress. The SAR framework encompasses guidelines targeting these previous aspects and provides students with helpful links to a range of resources based on these approaches (Appendix III).

### Depression

The current study found that depressive symptoms decreased significantly after implementing SAR guidelines. The improvement in depressive symptoms may be attributed to the design of the SAR framework, which enhances resilience at multiple levels (personal and environmental) throughout the various assessment stages (pre, during, and post) [3, 50]. The SAR framework’s “assessment preparation” and “student reflection” guidelines aim to improve personal assessment behavior (Appendix III). During SAR implementation, students were advised to prepare thoroughly for the exam and were given strategies for improving self-care and reducing test anxiety. Furthermore, the other SAR guidelines, particularly “assessment direction” and “assessment experience,” focus on the assessment organizational process to boost motivation and self-efficacy. These can lead to a reduction in “rumination,” a cognitive response style that consists of a vicious cycle of ruminating on negative thoughts [72]. Regarding this quantitative finding, course coordinators supported it by observing and reporting that mental health improved after SAR implementation (Table 5).

The current study is comparable to a randomized controlled trial confined to a single medical school in which researchers used a workshop-based intervention to promote the mental health of students, and the results demonstrated a significant reduction in depressive symptoms [67]. Furthermore, this study is consistent with Kunzler, Helmreich [59] systematic review, which found that resilience interventions result in improving depression with a low effect size. It is worth noting that the systematic review by Cleary, Kornhaber [58] revealed that some interventional studies improved depression, but not resilience. They attributed this outcome to the nature of the intervention, which focused on specific psychological problems, such as depression, while ignoring other crucial constructs, such as resilience. The current intervention addressed both concepts—depression and resilience—demonstrating its strength as a comprehensive and holistic approach.

### Burnout

Three types of burnout were measured in the current study—personal, work (academic), and client (patients). Personal and work-related (academic) burnout were significantly reduced by the use of SAR guidelines, which is consistent with the findings of two RCTs that found a significant decline in burnout following resilience intervention [73, 74]. This could be explained by the reciprocal mediating effect between resilience and burnout [4, 75, 76]. Moreover, the SAR guidelines were intended to improve personal and occupational factors (academic environment). SAR includes several guidelines aimed at personal factors, such as self-efficacy and motivation, as well as guidelines aimed at mitigating the negative effects of the academic environment, particularly the exam-related environment (Appendix III). As a result of implementing the SAR guidelines (personal and academic environment), resilience increased while burnout declined.

The factor that contributes to burnout reduction is the use of SAR guidelines at the process and organizational levels of an assessment (pre, intra, and post). Multiple meta-analyses have found that organizationally targeted interventions have a greater effect size than individually targeted interventions [8, 77–79]. This further supports the argument that burnout is likelier to be caused by organizational deficiencies than by individual factors [8, 77]. This effect is clearly described by medical educators who observed that students were significantly more engaged in clinical rotations when SAR was utilized than in rotations when it was not (Table 5).

Client-related burnout did not differ significantly between pre- and post-intervention measures. This could be explained by the fact that participants who had just begun their clinical rotation lacked the full authority to interact with patients and make clinical decisions. These actions were performed by clinicians, so students were not yet exposed to hospital-related burnout [8, 10].

### Correlation among study tools

The correlation analysis of study tools in pre- and post-intervention elucidates the dynamic interplay between resilience and psychological stress indicators such as depression, anxiety, stress, and burnout among participants before and after the intervention. We discovered a significant negative correlation between resilience and these indicators, highlighting resilience’s critical role in protecting against psychological distress. Such findings are consistent with prior research by Hayat, Choupani [51], Putwain, Becker [80], which suggest that enhancing resilience can significantly mitigate the adverse effects of stress, anxiety, and depression.

The practical implications of these insights are profound. They suggest that interventions aimed at bolstering resilience may be effective strategies for reducing psychological distress. In educational settings, this could translate to a resilience-built-in system designed to equip students with coping mechanisms, thereby enhancing their well-being and academic performance.

Regarding the strong correlation of anxiety, depression, stress, and burnout that was noticed in this study, a previous systematic review by Koutsimani, Montgomery [78] found that such psychological constructs are highly correlated even when using different scales.

Nevertheless, the current study has several strengths, one of which is that it used an experimental design with pre- and post-intervention evaluations. The timing of the study intervention was optimal, as it occurred in the middle of the academic year when stress levels were at their peak [7]. This underscores its effectiveness. Moreover, it was evident that these guidelines are user-friendly and self-explanatory. In addition to providing a system-integrated approach to promoting resilience, the study also includes tools for assessing resilience levels along with other counteracting factors.

On the other hand, the study has some limitations. One of these is that a comparative control group is impractical because preventing intervention leakage cannot be guaranteed in a single-institution study. Additional research that includes a control group is necessary to evaluate the efficacy of the SAR framework. Another challenge was that follow-up measurements were not conducted due to logistical constraints. After implementing the intervention, multiple measurements should be taken in a future study. Additionally, qualitative feedback was gathered from five course coordinators, rendering the sample size insufficient for achieving saturation in a qualitative study. Future research should aim to include an acceptable sample size for the qualitative evaluation of the study. Finally, it was limited to a single institution and the sample size is small; further study including multicenter research with large sample size is suggested.

Based on the findings of the current study, the researchers propose several recommendations. First, it is strongly recommended that the SAR guidelines be incorporated into medical and HPE curricula daily due to their clarity, practicability, and feasibility. Teachers in the medical and HPE fields should be encouraged to utilize them and be provided training to ensure their proper application. Second, in the “new normal” following the COVID-19 pandemic and the massive transformations it occasioned, nearly all medical schools have adopted blended learning. It is feasible to apply the SAR framework with this modification. E-learning platforms are fertile ground for employing SAR guidelines and their associated resources, and students would have unlimited access to them. Additionally, these resources are simple to expand and update regularly. Finally, the nature of SAR guidelines is not limited to medical and HPE students; they can be applied to students in other disciplines in higher education, and even to students in secondary and elementary schools.

## Conclusion

Resilience is a crucial attribute for students in the medical and health professions as it fosters the traits that graduates need to face future adversities. The current study presents a distinctive intervention using a quasi-experimental design with one group pre- and post-test for fostering resilience while simultaneously practicing the assessment. It uses what is called systematic assessment for resilience (SAR) to enhance resilience through a holistic approach across multiple levels. Comparing the results of pre- and post-intervention measurements yielded astounding results and showed significant improvement in resilience and a significant reduction in counteracting psychological parameters, such as stress, anxiety, depression, and burnout; thus, this study provides evidence-based guidance on how to promote resilience within an educational setting. By using this novel approach, the SAR framework, this research opens a new horizon for nurturing the mental well-being of future doctors who will provide better healthcare services to ensure patient safety. Future research is needed to monitor the impact of the SAR framework over time and broaden the scope of the intervention by including other HPE fields and students in higher education.

### Electronic supplementary material

Below is the link to the electronic supplementary material.

Supplementary Material 1

Supplementary Material 2

Supplementary Material 3

Supplementary Material 4

## Data Availability

Please email the corresponding author for a link to the de-identified datasets. However, due to privacy concerns, the in-depth interview transcripts are unavailable to the general public.

## Abbreviations

ARS: Academic Related Stressors
CBI: Copenhagen Burnout Inventory
CI: Confidence interval
DASS-21: Depression, Anxiety and Stress Scale 21
df: Degree of freedom
DOCEE: Direct Observation of the Clinical Examination Encounter
HPE: Health Professions Education
IQR: Inter Quartile Range
MeRS: Medical Professionals Resilience Scale
MSSQ: Medical Students Stressor Questionnaire
RCT: Randomized Control Trials
SAR: Systematic Assessment for Resilience
SD: Standard deviation
SPSS: Statistical Package for Social Sciences
