# Supplementary material for: The impact of the Systematic Assessment for Resilience (SAR) framework on students’ resilience, anxiety, depression, burnout, and academic-related stress: a quasi-experimental study
Source: BMC Med Educ. 2024 May 7;24:506. doi: 10.1186/s12909-024-05444-9 (PMC11077819; doi:10.1186/s12909-024-05444-9)

APPENDIX I: The training workshop

**Objectives**

- To highlight on the common mental health problems among medical students
- To recognize the concept of resilience and its importance for medical students
- To discuss the he places of resilience in the assessment process
- To discuss the SAR framework and how it can be applied.

**Timetable**

| Time | Topics/sections |
| --- | --- |
| 09:30 – 09:45 | Welcoming and pre-survey |
| 09:45 – 10:00 | Background |
| 10:00 – 10:30 | Resilience |
| 10:30 – 11:00 | How can we promote resilience in students' assessment? |
| 11:00 – 12:00 | Group activity |
| 12:00 – 12:30 | Break |
| 12:30 – 2:00 | Group presentation |
| 02:00 – 2:30 | SAR guidelines with example |
| 2:30 – 3:00 | Feedback, invitation and closing |

The PowerPoint materials are available here: shorturl.at/ilS49


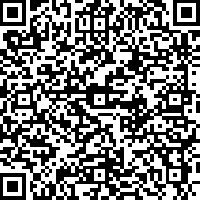

Supplement: Supplementary file 1 — Supplementary Material 1 [file 12909_2024_5444_MOESM1_ESM.docx]
