# Supplementary material for: The impact of the Systematic Assessment for Resilience (SAR) framework on students’ resilience, anxiety, depression, burnout, and academic-related stress: a quasi-experimental study
Source: BMC Med Educ. 2024 May 7;24:506. doi: 10.1186/s12909-024-05444-9 (PMC11077819; doi:10.1186/s12909-024-05444-9)
Supplement: Supplementary file 2 — Supplementary Material 2 [file 12909_2024_5444_MOESM2_ESM.docx]

APPENDIX II: The assignment for group work during the workshop

Thank you for your initial response. Now, the task will start as:

- **GROUPING**: You will be divided into groups.
- **REFLECT**: Individually, you are required to reflect on your initial response.
- **DISCUSS**: Then, discuss with your group this reflection.
- **SHARE**: Each group is given time to share the consensus about the application of these strategies to other groups.

| **Assessment phase** | **Categories of resilience** | **Resilience strategies** | **Your initial response** | | **If yes, how did you apply it?** | **If no, how could you apply it?** | **Other remark (if any)** |
| --- | --- | --- | --- | --- | --- | --- | --- |
|  |  |  | **Yes** | **No** |  |  |  |
| **During planning** | Assessment direction | Do you share students with assessment mapping/blueprint? |  |  |  |  |  |
|  |  | Do you share students with assessment rubric? |  |  |  |  |  |
|  |  | Do you brief students on the overall assessment coverage? |  |  |  |  |  |
|  |  | Do you establish a briefing session before exam? |  |  |  |  |  |
|  |  | Do you familiarize students with assessment methods? |  |  |  |  |  |
|  | Assessment preparation | Do you advise students about time management and other study skills? |  |  |  |  |  |
|  |  | Do you direct students for good materials for revision (e.g. textbooks, websites**, ..**etc)? |  |  |  |  |  |
|  |  | Do you advise students on exam preparation skills? |  |  |  |  |  |
|  |  | Do you provide students with strategies to reduce test anxiety? |  |  |  |  |  |
| **During education process** | Assessment experience | Do you increase frequency of formative assessment? |  |  |  |  |  |
|  |  | Do you have targeted mock exams? |  |  |  |  |  |
|  |  | Do you use collaborative assessment? |  |  |  |  |  |
|  |  | Do you use open book exam? |  |  |  |  |  |
|  |  | Do you use peer assessment? |  |  |  |  |  |
| **During exam day** | Examiner focus | Do you establish non-threatening environment during exam: smiling face, welcoming, professional behaviour, rapport, sense of humour? |  |  |  |  |  |
|  | Student reflection | Do you use frequent feedback as examiner to examinees? |  |  |  |  |  |
|  |  | Do you add free space/window for students to write/say self-reflection? |  |  |  |  |  |
