# Supplementary material for: The impact of the Systematic Assessment for Resilience (SAR) framework on students’ resilience, anxiety, depression, burnout, and academic-related stress: a quasi-experimental study
Source: BMC Med Educ. 2024 May 7;24:506. doi: 10.1186/s12909-024-05444-9 (PMC11077819; doi:10.1186/s12909-024-05444-9)
Supplement: Supplementary file 3 — Supplementary Material 3 [file 12909_2024_5444_MOESM3_ESM.docx]

APPENDIX III: The SAR supplementary materials for medical educators

**Using Systemic Assessment to promote Resilience (SAR): a practical guide**

| **Assessment phase** | | **Resilience strategies** | **Example** | **Methods of sharing/distribution** |
| --- | --- | --- | --- | --- |
| **Pre - course** | Assessment Direction | Sharing of assessment mapping/blueprinting whenever applicable | General blueprint | - Course booklet - Electronic platform (e.g. Blackboard) |
|  |  | Sharing of assessment rubric in modalities whenever applicable | Rubric | - Course booklet - Electronic platform (e.g. Blackboard) |
|  |  | Briefing on the overall assessment coverage | Overall assessment briefing | - Class session - Electronic platform (e.g. Blackboard) |
|  |  | Establishing a briefing session before exam | Before exam briefing | - Class session - Electronic platform (e.g. Blackboard) |
|  |  | Familiarizing students with assessment methods | Familiarizing assessment methods | - Class session - Electronic platform (e.g. Blackboard) |
|  | Assessment preparation | Advising students about time management and study skills | - Links to relevant materials (YouTube, Websites … etc) - Discussion or webinar - <https://youtu.be/MR7e1kPp_>[ys](https://youtu.be/MR7e1kPp_ys) | - Electronic platform (e.g. Blackboard) - Text advice through WhatsApp |
|  |  | Directing students for good material for revision | - Good handouts for revision - Concise reference books | - Electronic platform (e.g. Blackboard) |
|  |  | Advising students on exam skills | - Links to relevant materials (YouTube, Websites … etc) - Discussion or webinar | - Electronic platform (e.g. Blackboard) - Text advice through WhatsApp |
|  |  | Providing strategies for students to reduce test anxiety | - Advice about personal/selfcare (nutrition, sport, sleep) - Advice about relaxation techniques (deep breathing) - Discussion about these strategies - <https://youtu.be/>[4PgEIlewf7Y](https://youtu.be/4PgEIlewf7Y) | - Electronic platform (e.g. Blackboard) - Text advice through WhatsApp |
| **During - course** | Assessment Experience | Increasing frequency of formative assessment | More formative assessment |  |
|  |  | Encourage to have targeted mock exams | Mock exam |  |
|  |  | Promoting/encouraging collaborative assessment | Collaborative assessment <https://youtu.be/RNOC2OHP6Vs> | - Class session - Electronic platform (e.g. Blackboard) |
|  |  | Promoting open book exam | Open book exam  <https://youtu.be/-xVwAdCgdlQ> | - Class session - Electronic platform (e.g. Blackboard) |
|  |  | Using peer assessment | Peer assessment  <https://youtu.be/2hRu5i-gfXo> | - Class session - Electronic platform (e.g. Blackboard) |
| **On exam day** | Examiner focus | Establishing non-threatening environment during exam | - smiling face, - welcoming, - professional behaviour, - rapport, - sense of humour | - on the exam day |
|  | Student reflection | Increasing feedback to examinee | <https://youtu.be/s8Jl-8JLxdo> | - on the exam day |
|  |  | Adding free space/window for self-reflection | <https://youtu.be/SntBj0FIApw> | - on the exam day |
