# Supplementary material for: The impact of the Systematic Assessment for Resilience (SAR) framework on students’ resilience, anxiety, depression, burnout, and academic-related stress: a quasi-experimental study
Source: BMC Med Educ. 2024 May 7;24:506. doi: 10.1186/s12909-024-05444-9 (PMC11077819; doi:10.1186/s12909-024-05444-9)
Supplement: Supplementary file 4 — Supplementary Material 4 [file 12909_2024_5444_MOESM4_ESM.docx]

**Appendix IV: Study tools and their scoring**

Table of Contents

[Resilience using Medical Professionals Resilience Scale (MeRS) (1) 2](#_Toc159321813)

[Academic related stress using MSSQ (2) 4](#_Toc159321814)

[Depression and Anxiety using DASS 21 (3) 5](#_Toc159321815)

[Burnout using Copenhagen Burnout Inventory (4) 6](#_Toc159321816)

[References: 8](#_Toc159321817)

# **Resilience using Medical Professionals Resilience Scale (MeRS) (1)**

1. **Instruction to students:**

From the listed items below, please rate by selecting what you feel according to the following: 1 = Strongly disagree, 2 = Disagree, 3 = Agree, 4 = Strongly agree

| **Resilience domain** | **No.** | **Item Statement** | **Scales** | | | |
| --- | --- | --- | --- | --- | --- | --- |
|  |  |  | **Strongly**  **disagree** | **Disagree** | **Agree** | **Strongly**  **agree** |
| **Growth**  **(number of items = 15)** |  | I can succeed if I keep trying | 1 | 2 | 3 | 4 |
|  |  | I believe everything happens for a reason | 1 | 2 | 3 | 4 |
|  |  | When I face new situations, I will learn from it | 1 | 2 | 3 | 4 |
|  |  | I believe there is a wisdom behind everything in life | 1 | 2 | 3 | 4 |
|  |  | I believe every problem comes with a solution | 1 | 2 | 3 | 4 |
|  |  | I seek help to achieve my goals if necessary | 1 | 2 | 3 | 4 |
|  |  | I believe by helping others, I am helping myself too | 1 | 2 | 3 | 4 |
|  |  | I have goals to achieve | 1 | 2 | 3 | 4 |
|  |  | I believe good planning is a key to success | 1 | 2 | 3 | 4 |
|  |  | I believe self-motivation will change the final outcome | 1 | 2 | 3 | 4 |
|  |  | I believe hard work really pays off in the end | 1 | 2 | 3 | 4 |
|  |  | I am aware of my strengths and abilities | 1 | 2 | 3 | 4 |
|  |  | I am positive I will be successful in the future | 1 | 2 | 3 | 4 |
|  |  | When my work is criticized, I cope positively by trying harder the next time | 1 | 2 | 3 | 4 |
|  |  | Believing in myself helps me to face any difficulties | 1 | 2 | 3 | 4 |
| **Involvement**  **(number of items = 12)** |  | I am firm with my stand | 1 | 2 | 3 | 4 |
|  |  | I can adapt to change at work situations | 1 | 2 | 3 | 4 |
|  |  | People always believe in me to make difficult decision | 1 | 2 | 3 | 4 |
|  |  | I am comfortable working in new environment | 1 | 2 | 3 | 4 |
|  |  | I spend my life doing something great | 1 | 2 | 3 | 4 |
|  |  | I have good coping skills when dealing with stress | 1 | 2 | 3 | 4 |
|  |  | I am proud of my own accomplishments | 1 | 2 | 3 | 4 |
|  |  | I become a stronger person when facing difficulties at work | 1 | 2 | 3 | 4 |
|  |  | I always give my best at work | 1 | 2 | 3 | 4 |
|  |  | I feel energetic doing my work even in difficult situations | 1 | 2 | 3 | 4 |
|  |  | I can maintain interest in my work | 1 | 2 | 3 | 4 |
|  |  | My colleagues can always rely on me | 1 | 2 | 3 | 4 |
| **Control** |  | I can stay calm in hard situations | 1 | 2 | 3 | 4 |
|  |  | I can handle unpleasant feelings | 1 | 2 | 3 | 4 |
|  |  | I always try to stay calm in any situation | 1 | 2 | 3 | 4 |
|  |  | I can control my anger | 1 | 2 | 3 | 4 |
|  |  | I am in control of my surroundings | 1 | 2 | 3 | 4 |
|  |  | I am good at adapting myself to different situations | 1 | 2 | 3 | 4 |
| **Resourceful**  **(number of items = 4** |  | I know who to talk to when I have a problem | 1 | 2 | 3 | 4 |
|  |  | I know where to go if I need help | 1 | 2 | 3 | 4 |
|  |  | I always have someone by my side when I have problems | 1 | 2 | 3 | 4 |
|  |  | I figure out ways to solve my problems by talking about them | 1 | 2 | 3 | 4 |

1. **Scoring guide:**

**Domain for Each Item**

| Domain | Item Number | Scoring |
| --- | --- | --- |
| Growth | 1-15 | Sum of scores for all items |
| Involvement | 16-27 | Sum of scores for all items |
| Control | 28-33 | Sum of scores for all items |
| Resourceful | 34-37 | Sum of scores for all items |

**MeRS Scoring and respected Level of resilience competency**

| MeRS domain  (number of items) | Level of resilience competency (Total score) | | |
| --- | --- | --- | --- |
|  | **Developing level (low)** | **Established level (moderate)** | **Exceptional level (high)** |
| Growth (15) | 15 - 27 | 28-47 | 48 - 60 |
| Involvement (12) | 12 – 21 | 22 - 38 | 39 - 48 |
| Control (6) | 6 – 11 | 12 - 18 | 19 - 24 |
| Resourceful (4) | 4 – 7 | 8 - 12 | 13 - 16 |
| Global score (37)* | 37 - 66 | 67 - 118 | 119 - 148 |

*Global score: sum of scores for all domains

**Definitions of Each Level of resilience competency**

| MeRS domain | Level of resilience competency (Definition) | | |
| --- | --- | --- | --- |
|  | **Developing level (low)** | **Established level (moderate)** | **Exceptional level (high)** |
| Control | Less composed and controlled under stressful adversity | Acceptably composed and controlled under stressful adversity | Highly composed and controlled under stressful adversity |
| Resourceful | Less able to find appropriate solutions from available resources to deal with adversity | Acceptably able to find appropriate solutions from available resources to deal with adversity | Highly able to find appropriate solutions from available resources to deal with adversity |
| Involvement | Less committed to deal with adversity | Acceptably committed to deal with adversity | Highly committed to deal with adversity |
| Growth | Less able to keep growing and bouncing back stronger from adversity | Acceptably able to keep growing and bouncing back stronger from adversity | Highly able to keep growing and bouncing back stronger from adversity |

# **Academic related stress using MSSQ (2)**

**Note:** The following 13 items related to academic stress (academic-related stress, ARS) were derived from the Medical Student Stress Questionnaire (MSSQ) (2).

1. **Instruction for students:**

From the listed items below, please rate by selecting what you feel according to the following: 0 = causing no stress at all, 1 = causing mild stress, 2 = causing moderate stress, 3 = causing high stress, 4 = causing severe stress

| Items | 0 | 1 | 2 | 3 | 4 |
| --- | --- | --- | --- | --- | --- |
| 1. Tests/examinations |  |  |  |  |  |
| 1. Quota system in examination |  |  |  |  |  |
| 1. Need to do well (self-expectation) |  |  |  |  |  |
| 1. Heavy workload |  |  |  |  |  |
| 1. Falling behind in reading schedule |  |  |  |  |  |
| 1. Not enough medical skill practice |  |  |  |  |  |
| 1. Learning context – full of competition |  |  |  |  |  |
| 1. Having difficulty understanding the content |  |  |  |  |  |
| 1. Getting poor mark |  |  |  |  |  |
| 1. Lack of time to review what have been learnt |  |  |  |  |  |
| 1. Unable to answer the questions from teachers |  |  |  |  |  |
| 1. Large amount of content to be learnt |  |  |  |  |  |
| 1. Unjustified grading process |  |  |  |  |  |

1. **Scoring guide:**

Scoring guide of ARS (2) is by sum the responses and divided by 13.

| Range | Interpretation |
| --- | --- |
| 0 – 1.00 | Mild stress |
| 1.01 – 2.00 | Moderate stress |
| 2.01 – 3.00 | High stress |
| 3.01 – 4.00 | Severe stress |

# **Depression and Anxiety using DASS 21 (3)**

**Note:** From the DASS 21 inventory (3), only the subsets pertaining to depression and anxiety, each consisting of 7 items, were included in the distributed measurement tools.

1. **Instruction for students:**

Please read each statement and circle a number 0, 1, 2 or 3 which indicates how much the statement applied to you over the past week. There are no right or wrong answers. Do not spend too much time on any statement.

| **Domain** | **Item code as found in DASS 21** | **Items** | **Scale** |
| --- | --- | --- | --- |
| Anxiety | A2 | I was aware of dryness of my mouth | 0 1 2 3 |
| Depression | D3 | I couldn't seem to experience any positive feeling at all | 0 1 2 3 |
| Anxiety | A4 | I experienced breathing difficulty (eg, excessively rapid breathing, breathlessness in the absence of physical exertion) | 0 1 2 3 |
| Depression | D5 | I found it difficult to work up the initiative to do things | 0 1 2 3 |
| Anxiety | A7 | I experienced trembling (eg, in the hands) | 0 1 2 3 |
| Anxiety | A9 | I was worried about situations in which I might panic and make a fool of myself | 0 1 2 3 |
| Depression | D10 | I felt that I had nothing to look forward to | 0 1 2 3 |
| Depression | D13 | I felt down-hearted and blue | 0 1 2 3 |
| Anxiety | A15 | I felt I was close to panic | 0 1 2 3 |
| Depression | D16 | I was unable to become enthusiastic about anything | 0 1 2 3 |
| Depression | D17 | I felt I wasn't worth much as a person | 0 1 2 3 |
| Anxiety | A19 | I was aware of the action of my heart in the absence of physical exertion (eg, sense of heart rate increase, heart missing a beat) | 0 1 2 3 |
| Anxiety | A20 | I felt scared without any good reason | 0 1 2 3 |
| Depression | D21 | I felt that life was meaningless | 0 1 2 3 |

1. **Scoring guide:**

Scores on the DASS-21 will need to be multiplied by 2 to calculate the final score.

|  | Depression | Anxiety |
| --- | --- | --- |
| Normal | 0 – 9 | 0 – 7 |
| Mild | 10 – 13 | 8 – 9 |
| Moderate | 14 – 20 | 10 – 14 |
| Severe | 21 – 17 | 15 – 19 |
| Extremely severe | 28+ | 20+ |

# **Burnout using Copenhagen Burnout Inventory (4)**

1. **Part one: Personal burnout**

**Definition:** Personal burnout is a state of prolonged physical and psychological exhaustion.

**Instruction for students:**

Please respond to the following question either by: Always, Often, Sometimes, Seldom, Never/almost never.

| Items | Always | Often | Sometimes | Seldom | | Never | |  |
| --- | --- | --- | --- | --- | --- | --- | --- | --- |
| 1. How often do you feel tired? |  |  |  | |  | |  | |
| 1. How often are you physically exhausted? |  |  |  | |  | |  | |
| 1. How often are you emotionally exhausted? |  |  |  | |  | |  | |
| 1. How often do you think:”Ican’t take it anymore”? |  |  |  | |  | |  | |
| 1. How often do you feel worn out? |  |  |  | |  | |  | |
| 1. How often do you feel weak and susceptible to illness? |  |  |  | |  | |  | |

**Scoring guide:**

- Always: 100. Often: 75. Sometimes: 50. Seldom: 25. Never/almost never: 0. Total score on the scale is the average of the scores on the items.
- If less than three questions have been answered, the respondent is classified as non-responder.

1. **Part two: Work-related burnout**

**Definition:** Work-related burnout is a state of prolonged physical and psychological exhaustion, which is perceived as related to the person’s work.

**Instruction for students:**

Please respond to the following question according to the following:

- The four first questions: To a very high degree, To a high degree, Somewhat, To a low degree, To a very low degree.
- The two last questions: Always, Often, Sometimes, Seldom, Never/almost never.

| Items | To a very high degree | To a high degree | Somewhat | To a low degree | To a very low degree |
| --- | --- | --- | --- | --- | --- |
| 1. Is your work emotionally exhausting? |  |  |  |  |  |
| 1. Do you feel burnt out because of your work? |  |  |  |  |  |
| 1. Does your work frustrate you? |  |  |  |  |  |
| 1. Do you feel worn out at the end of the working day? |  |  |  |  |  |
|  | **Always** | **Often** | **Sometimes** | **Seldom** | **Never** |
| 1. Are you exhausted in the morning at the thought of another day at work? |  |  |  |  |  |
| 1. Do you feel that every working hour is tiring for you? |  |  |  |  |  |
| 1. Do you have enough energy for family and friends during leisure time? |  |  |  |  |  |

**Scoring guide:**

- Scoring as for the first scale. If less than four questions have been answered, the respondent is classified as non-responder.

1. **Part three: Client-related burnout**

**Definition**: Client-related burnout is a state of prolonged physical and psychological exhaustion, which is perceived as related to the person’s work with clients*. *Clients, patients, social service recipients, elderly citizens, or inmates.

**Instruction for students:**

Please respond to the following question according to the following:

- The four first questions: To a very high degree, To a high degree, Somewhat, To a low degree, To a very low degree.
- The two last questions: Always, Often, Sometimes, Seldom, Never/almost never.

| Items | To a very high degree | To a high degree | Somewhat | To a low degree | To a very low degree |
| --- | --- | --- | --- | --- | --- |
| 1. Do you find it hard to work with clients? |  |  |  |  |  |
| 1. Do you find it frustrating to work with clients? |  |  |  |  |  |
| 1. Does it drain your energy to work with clients? |  |  |  |  |  |
| 1. Do you feel that you give more than you get back when you work with clients? |  |  |  |  |  |
|  | **Always** | **Often** | **Sometimes** | **Seldom** | **Never** |
| 1. Are you tired of working with clients? |  |  |  |  |  |
| 1. Do you sometimes wonder how long you will be able to continue working with clients? |  |  |  |  |  |

**Scoring guide:**

- Scoring as for the first scale. If less than four questions have been answered, the respondent is classified as non-responder.

# **References:**

1. 1. Rahman MA, Yusoff MSB, Roslan NS, Mohammad JA-M, Ahmad A. Development and validation of the medical professionals resilience scale. BMC Health Services Research. 2021;21(1):482. <https://doi.org/10.1186/s12913-021-06542-w>
2. 2. Yusoff MSB, Rahim AFA, Yaacob MJ. The development and validity of the Medical Student Stressor Questionnaire (MSSQ). ASEAN Journal of Psychiatry. 2010;11(1):231-5. Available at: <https://citeseerx.ist.psu.edu/document?repid=rep1&type=pdf&doi=4c7e3c6a2948bd424adec12e067eb1b9cd4d8f58>
3. 3. Zanon C, Brenner RE, Baptista MN, Vogel DL, Rubin M, Al-Darmaki FR, et al. Examining the Dimensionality, Reliability, and Invariance of the Depression, Anxiety, and Stress Scale–21 (DASS-21) Across Eight Countries. Assessment. 2020;28(6):1531-44. <https://doi.org/10.1177/1073191119887449>
4. 4. Kristensen TS, Borritz M, Villadsen E, Christensen KB. The Copenhagen Burnout Inventory: A new tool for the assessment of burnout. Work & Stress. 2005;19(3):192-207. <https://doi.org/10.1080/02678370500297720>
